# Arterial to end-tidal Pco_2_ difference during exercise in normoxia and severe acute hypoxia: importance of blood temperature correction

**DOI:** 10.14814/phy2.12512

**Published:** 2015-10-27

**Authors:** José Losa-Reyna, Rafael Torres-Peralta, Juan José González Henriquez, José A L Calbet

**Affiliations:** 1 Department of Physical Education, University of Las Palmas de Gran CanariaLas Palmas de Gran Canaria, Spain; 2 Research Institute of Biomedical and Health Sciences (IUIBS)Las Palmas de Gran Canaria, Canary Islands, Spain; 3 Department of Mathematics, University of Las Palmas de Gran CanariaLas Palmas de Gran Canaria, Spain

**Keywords:** Blood temperature, gas exchange, gradient, hypoxia, Pco_2_

## Abstract

Negative arterial to end-tidal Pco_2_ differences ((a-ET)Pco_2_) have been reported in normoxia. To determine the influence of blood temperature on (a-ET)Pco_2_, 11 volunteers (21 ± 2 years) performed incremental exercise to exhaustion in normoxia (Nx, P_I_o_2_: 143 mmHg) and hypoxia (Hyp, P_I_o_2_: 73 mmHg), while arterial blood gases and temperature (ABT) were simultaneously measured together with end-tidal Pco_2_ (P_E__T_co_2_). After accounting for blood temperature, the (a-ET) Pco_2_ was reduced (in absolute values) from −4.2 ± 1.6 to −1.1 ± 1.5 mmHg in normoxia and from −1.7 ± 1.6 to 0.9 ± 0.9 mmHg in hypoxia (both *P* < 0.05). The temperature corrected (a-ET)Pco_2_ was linearly related with absolute and relative exercise intensity, VO_2_, VCO_2_, and respiratory rate (RR) in normoxia and hypoxia (*R*^2^: 0.52–0.59). Exercise CO_2_ production and P_E__T_co_2_ values were lower in hypoxia than normoxia, likely explaining the greater (less negative) (a-ET)Pco_2_ difference in hypoxia than normoxia (*P* < 0.05). At near-maximal exercise intensity the (a-ET)Pco_2_ lies close to 0 mmHg, that is, the mean P_a_co_2_ and the mean P_E__T_co_2_ are similar. The mean exercise (a-ET)Pco_2_ difference is closely related to the mean A-aDO_2_ difference (*r* = 0.90, *P* < 0.001), as would be expected if similar mechanisms perturb the gas exchange of O_2_ and CO_2_ during exercise. In summary, most of the negative (a-ET)Pco_2_ values observed in previous studies are due to lack of correction of P_a_co_2_ for blood temperature. The absolute magnitude of the (a-ET)Pco_2_ difference is lower during exercise in hypoxia than normoxia.

## Introduction

The alveolar-to-arterial Po_2_ difference (A-aDO_2_, P_A_o_2_-P_a_o_2_) increases with exercise intensity in humans (Holmgren and Linderholm 1958; Dempsey et al. 1984), and to a greater extent in hypoxia than in normoxia (Torre-Bueno et al. 1985; Schaffartzik et al. 1992; Wagner 1992; Calbet et al. 2008). In contrast, both positive and negative arterial to end-tidal Pco_2_ ((a-ET)Pco_2_) values have been reported during exercise (Forster 1977; Gurtner 1977; Piiper 1986). It has been postulated that negative (a-ET)Pco_2_ differences could be in part due to measurement artifacts, such as loss of CO_2_ from blood samples, dilution with heparin solutions present in syringes, and underestimation of lung temperature (Scheid and Piiper 1980; Piiper 1986).

Although the (a-ET)Pco_2_ has been studied in healthy exercising humans (Whipp and Wasserman 1969; Jones et al. 1979; Robbins et al. 1990; Williams and Babb 1997) and patients with lung disease (Luft et al. 1979; Mahler et al. 1985; Liu et al. 1995), in none of these studies was the (a-ET)Pco_2_ calculation corrected to account for the increase of lung blood temperature during exercise.

Due to the high diffusivity of CO_2_, mean alveolar Pco_2_ (P_A_co_2_) is similar to the end capillary Pco_2_ in well-ventilated and perfused alveoli and hence, similar to P_a_co_2_ (Cerretelli and Di Prampero 1987). However, mean P_A_co_2_ and P_E__T_co_2_ may fluctuate differently during the respiratory cycle (Hlastala 1972), both at rest and during exercise (Dubois et al. 1952; Johnson et al. 2011). One of the main factors influencing P_E__T_co_2_ and hence (a-ET)Pco_2_, is the respiratory rate (Dubois et al. 1952; Hlastala 1972; Johnson et al. 2011). Compared to normoxia, during submaximal exercise in hypoxia pulmonary ventilation is increased by a combined elevation of tidal volume (VT) and respiratory rate (RR) (Paterson et al. 1987; Lundby et al. 2004; Calbet and Lundby 2009). In theory, for a given VO_2_, (a-ET)Pco_2_ should increase with greater ventilation. However, the effect of severe hypoxia on exercise (a-ET)Pco_2_ has not been assessed.

Therefore, the primary aim of this study was to examine the impact of: (1) blood temperature correction; and (2) severe hypoxia on the (a-ET)Pco_2_ difference during exercise in healthy subjects.

We hypothesized that: (1) correcting for blood temperature will reduce the absolute value of the (a-ET)Pco_2_ difference; and (2) the absolute value (a-ET)Pco_2_ difference will be lower during exercise in severe hypoxia than in normoxia, due to a greater impairment of pulmonary gas exchange during exercise in hypoxia.

## Methods

### General overview

This study was part of a larger project including several experiments designed to address the mechanisms limiting whole-body exercise performance in humans with assessment of central and local hemodynamics combined with measurements of oxygen transport, and pulmonary and muscle gas exchange (Calbet et al. 2015; González-Henriquez et al. 2015; Morales-Alamo et al. 2015). On the first visit to the laboratory, anthropometric measures and body composition analysis were performed. Thereafter, subjects reported to the laboratory on separate days to complete different incremental tests to exhaustion (see Respiratory variables below) in normoxia and hypoxia (Lode Excalibur Sport 925900, Groningen, The Netherlands). Subjects were requested to refrain from ingesting caffeine- and taurine-containing drinks and from exercise 24 h before the experiments.

## Subjects

Eleven healthy men participated in these studies. Their mean ± SD age, height, weight, percentage of body fat, and maximal oxygen uptake (VO_2_max) were 21.5 ± 2.0 years, 173.8 ± 8.0 cm, 72.3 ± 9.3 kg, 16.1 ± 4.9%, and 3.621 ± 0.326 L min^−1^, respectively. Before any experimental procedure, subjects received full oral and written information about the experiments. The study was performed in accordance with the Helsinki Declaration and was approved by the Ethical Committee of the University of Las Palmas de Gran Canaria (CEIH-2010-01).

### Catheterization and preparation for the experiments

Both femoral veins and one femoral artery were catheterized under local anesthesia (2% lidocaine), as previously reported (Calbet et al. 2006). In the right femoral vein, a 16G catheter was inserted 3-cm below inguinal ligament and advanced 12–13 cm distally (Arrow ES-04306). This catheter was used for saline ice-cold injection to measure the leg blood flow (LBF) by thermodilution (Andersen and Saltin 1985). In the same femoral vein, a thermodilution catheter (PV2014L16N, Pulsion Medical Systems AG, Munich, Germany) was inserted 2 cm below the inguinal ligament and advanced 12 cm cranially. This catheter was used to measure the temperature of the blood in the femoral vein. The same type of catheter was also inserted into the right femoral artery and used to measure blood pressure and femoral artery blood temperature. A final 20G catheter was inserted into the contralateral femoral vein from 2 cm below the inguinal ligament and advanced 12 cm in the direction toward the heart (Arrow ES-04150), and used for sampling femoral vein blood. All catheters were doubly sutured to the skin at the insertion point.

The two thermistors were connected to the temperature conditioning and processing boxes (Flemming Jessen Engineering, Copenhagen, Denmark), and the right femoral artery and vein catheters to blood pressure bridge amplifiers (ML-117, ADInstruments, Sydney, Australia).

An electrocardiogram (ECG) was displayed on a monitor during catheterization and the rest of the experimental procedures for safety reasons. The ECG, blood pressure, and the temperature registered by the thermistor, as well as the infusate temperature were recorded simultaneously with the data acquisition system (Power Lab ML880, ADInstruments, Bella Vista, Australia).

### Exercise protocol

On the experimental day, subjects reported to the laboratory at 07.00 in fasted conditions. After catheterization, subjects were assigned randomly to either an incremental exercise test until volitional exhaustion in normoxia (P_I_o_2_: ∼143 mmHg) or hypoxia (P_I_o_2_: ∼73 mmHg, Altitrainer200, SMTEC, Switzerland). The test in normoxia started at 80 W with load increments of 30 W every 2 min. The test in hypoxia started at 60 W with load increments of 20 W every 2 min until exhaustion (Exh1). At exhaustion, the subjects were rapidly switched to breathe room air (normoxia) and were requested to continue exercising at the same load for 2 min, and then the load was increased by 20 W every 2 min until exhaustion (Exh2). The tests were separated by 90 min rest. After the second test, a lunch break and a 120 min resting period were followed. Thereafter, the incremental exercise in hypoxia was repeated.

### Blood sampling

Blood samples were drawn simultaneously from the arterial and venous femoral catheters over a 10-sec period during the last minute of the step of each workload. The sampling period was then aligned with the respective respiratory data, assuming a circulating time of ∼10 sec (Calbet and Boushel 2015). Blood gases and hemoglobin concentrations were determined immediately after collection (ABL90, Radiometer, Copenhagen, Denmark). Uncorrected blood gases were expressed at 37°C. Arterial blood gasses and pH were corrected for blood temperature, using the arterial thermistor. Arterial Po_2_ and pH were corrected using Severinghaus equations (Severinghaus 1979), while Pco_2_ was corrected, using the equation Pco_2_tc = Pco_2(37)_*(10^0.021*(*T*-37)) according to Siggaard-Andersen (Siggaard-Andersen 1974), where Pco_2_tc is the temperature-corrected Pco_2_, Pco_2(37)_ is the Pco_2_ measured at 37°C, and *T* is the arterial blood temperature.

### Respiratory variables

Respiratory variables were recorded continuously with a metabolic cart (Vmax N29; Sensormedics, California), calibrated prior to each test according to the manufacturer instructions with high-grade calibration gases (Carburos Metálicos, Las Palmas de Gran Canaria). Respiratory variables were analyzed breath-by-breath and averaged every 10 sec during the incremental exercise tests. Then, the respiratory data were aligned with the appropriate blood sample, assuming a 10-sec shift between pulmonary gas exchange and arterial blood gases.

### Statistical analysis

Data are expressed as the mean ± standard deviation (SD) unless otherwise stated. Random-effects regression models were applied for data analysis. The random intercepts and slopes models were compared. The random intercepts models fit better into the data in all cases. Intercept and experimental error were assumed to have a Gaussian distribution. The model was estimated, using the restricted maximum likelihood method. For the goodness of fit, the conditional Nakagawa and Schielzeth’s *R*^2^_GLMM_ was used (Nakagawa and Schielzeth 2013). In addition, near-maximal exercise (a-ET)Pco_2_ values were compared between normoxia and hypoxia, using a paired Student t-test. The relationship between the mean (a-ET)Pco_2_ and the mean A-aDO_2_ was tested with linear regression analysis. *P* ≤ 0.05 was considered significant. Analysis was performed using a commercially available software package (SPSS version 15.0, SPSS, Inc., Chicago, Illinois) and The R Project for Statistical Computing version 3.2.0.

## Results

The mean responses of the respiratory variables to both exercise conditions are reported in Table1. The mean of all P_E__T_co_2_ measured values (submaximal and maximal exercise conditions) was 3.2 ± 2.3 mmHg higher than P_a_co_2_ (37.7 ± 5.8 mmHg and 33.5 ± 4.2 mmHg, respectively, *P* < 0.01). After temperature correction, the mean P_a_co_2_ increased to 34.9 ± 4.3 mmHg, consequently the (a-ET)Pco_2_ was increased from −3.2 ± 2.3 to −1.8 ± 2.1 mmHg (*P* < 0.01). This correction of the P_a_co_2_ value for arterial blood temperature accounted for 44% of the measured (a-ET)Pco_2_. The effect of the temperature correction on the magnitude of the (a-ET)Pco_2_ was greater during exercise in normoxia than hypoxia, and increased with exercise intensity (Table2). After accounting for blood temperature the (a-ET)Pco_2_ was increased from −4.2 to −1.1 mmHg in normoxia, and from −1.7 to 0.9 mmHg in hypoxia (Table2).

**Table 1:** Respiratory variables and arterial blood pH during exercise in normoxia (P_I_o_2_: ∼143 mmHg) and hypoxia (P_I_o_2_: ∼73 mmHg)

	*n*	Intensity (%Wpeak)	Intensity (watts)	VO_2_ (L·min^−1^)	VCO_2_ (L·min^−1^)	V_E_ (L·min^−1^)	RR (breaths·min^−1^)	V_T_ (mL)	P_E__T_co_2_ (mmHg)	P_E__T_o_2_ (mmHg)	P_a_co_2_ (mmHg)	P_a_co_2_tc (mmHg)	Arterial pH tc
Normoxia													
Mean	9	28.5	82.2	1.58	1.39	35.9	21.1	1738	43.7	94.1	39.0	39.48	7.393
SD		1.7	6.7	0.14	0.24	6.9	4.7	301	3.7	5.9	3.8	3.73	0.022
Mean	6	69.4	215	3.18	3.37	88.4	37.3	2366	43	102.1	38.6	40.37	7.304
SD		1.3	25.1	0.26	0.28	11.4	3.6	217	4	3.1	3.1	3.29	0.031
Mean	11	78.3	225.5	3.25	3.55	99.4	39.7	2516	40.4	105.5	35.6	37.72	7.282
SD		2	34.5	0.38	0.48	13.4	6.2	215	2.5	2	1.7	2.2	0.038
Mean	11	89.2	256.4	3.54	4.03	124.7	48.1	2594	37.1	109.7	32.8	34.99	7.259
SD		1.0	36.7	0.31	0.4	17.7	4.8	258	3.2	3.1	1.6	1.82	0.044
Mean	9	100	290	3.62	4.24	143.2	59.1	2445	35.3	112.6	31.7	34.18	7.221
SD		0	42.4	0.39	0.47	19.4	7.3	374	2.1	3	2.9	3.06	0.041
Hypoxia													
Mean	9	44.4	82.2	1.62	1.8	55.5	26.6	2118	34.4	43.2	32.3	32.69	7.441
SD		5.0	6.7	0.18	0.14	6.9	4.6	303	3.2	0.8	1.9	2.04	0.035
Mean	7	70.7	125.7	1.96	2.37	79.2	36.7	2176	31.6	45.8	30.3	31.05	7.391
SD		4.8	27.6	0.31	0.48	18.7	9.4	222	3.2	2.2	2.5	2.42	0.04
Mean	7	79.2	150	2.3	2.92	98.8	41.7	2372	30.4	47.4	29.6	30.55	7.335
SD		2.6	30	0.3	0.45	20.1	8.2	171	3.3	2.2	2.9	2.83	0.047
Mean	6	88.8	160	2.33	2.95	104.7	48.4	2167	28.5	49	28.2	29.39	7.317
SD		1.3	21.9	0.22	0.43	12.3	4.6	205	2	1.4	2.1	2.03	0.064

*n* = number of subjects.

**Table 2:** Intensity (%Wpeak), arterial blood temperature (°C) and arterial-to-end-tidal Pco_2_ difference ((a-ET)Pco_2_) (mmHg) during exercise in normoxia (P_I_o_2_: ∼143 mmHg) and hypoxia (P_I_o_2_: ∼73 mmHg) without and with blood temperature correction (tc)

Intensity (%Wpeak)			Arterial temperature (°C)			(a-ET)Pco_2_ (mmHg)			(a-ET)Pco_2_ (tc) (mmHg)		
Mean	SD	*n*	Mean	SD	Range	Mean	SD	Range	Mean	SD	Range
Normoxia											
28.5	1.7	9	37.3	0.5	36.4–38.0	−4.8	1.8	(−8.1) to (−2.8)	−4.2	1.6	(−7.1) to (−2.3)
69.4	1.3	6	37.9	0.4	37.4–38.6	−4.4	1.4	(−6.2) to (−2.9)	−2.6	1.3	(−4.2) to (−0.9)*
78.3	2.0	11	38.2	0.5	37.5–39.0	−4.8	1.8	(−8.0) to (−1.9)	−2.7	1.8	(−6.2) to (0.8)*
89.2	1.0	11	38.4	0.5	37.5–39.1	−4.3	2.4	(−9.6) to (−0.3)	−2.1	2.2	(−6.5) to (2.2)*
100.0	0.0	9	38.5	0.4	37.9–39.3	−3.6	1.6	(−5.6) to (−0.8)	−1.1	1.5	(−3.4) to (0.8)*
Hypoxia											
44.4	5.0	9	37.2	0.4	36.4–37.7	−2.0	1.8	(−4.9) to (0.8)	−1.7	1.6	(−4.1) to (0.1)
70.7	4.8	7	37.5	0.5	36.8–38.1	−1.3	1.3	(−2.4) to (1.1)	−0.6	1.3	(−2.3) to (1.6)
79.2	2.6	7	37.7	0.3	37.1–38.0	−0.8	1.0	(−2.6) to (0.8)	0.1	1.0	(−1.2) to (1.7)*
88.8	1.3	6	37.9	0.2	37.6–38.2	−0.3	0.8	(−1.7) to (0.4)	0.9	0.9	(−0.6) to (2.0)*

*n* = number of subjects per intensity.

* *P* < 0.05 vs. uncorrected (a-ET)Pco_2_.

Random-effects regression analyses between (a-ET)Pco_2_ and respiratory variables are shown in Table3. After temperature correction, (a-ET)Pco_2_ was linearly related to absolute and relative exercise intensity, VO_2_, VCO_2_, and RR in normoxia and hypoxia (Table3). In normoxia, there was also a linear relationship between (a-ET)Pco_2_ with VT and A-aDO_2_tc. The intercept of the linear relationship between (a-ET)Pco_2_ and the absolute load was significantly higher in hypoxia than in normoxia, while the slopes were similar. Likewise, for a given respiratory rate, (a-ET)Pco_2_ was higher in hypoxia than in normoxia (Table3). Since the intercepts and slopes of the linear relationship between (a-ET)Pco_2_ and the relative intensity were not significantly different between normoxia and hypoxia, a combined random-effects regression equation (eq. 1) was generated:

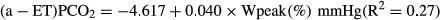
1

**Table 3:** Random-effects regression analysis between the arterial-to-end-tidal Pco_2_ (mmHg) difference ((a-ET)CO_2_) corrected for arterial blood temperature, exercise intensity and respiratory variables during exercise in normoxia (P_I_o_2_: ∼143 mmHg) and hypoxia (P_I_o_2_: ∼73 mmHg)

	Normoxia		Hypoxia		Normoxia (*P* values)		Hypoxia (*P* values)		Nox vs. Hyp	Nox vs. Hyp	*R* ^2^
	Intercept ± SE	Slope ± SE	Intercept ± SE	Slope ± SE	I	S	I	S	Ic	Sc	
Intensity (w)	−5.364 ± 0.669	0.013 ± 0.003	−2.712 ± 1.021	0.018 ± 0.008	0.000	0.000	0.009	0.020	0.025	0.54	0.54
Intensity (%Wpeak)	−5.531 ± 0.655	0.04 ± 0.008	−4.436 ± 1.085	0.058 ± 0.015	0.000	0.000	0.000	0.000	0.38	0.30	0.56
VO_2_ (L·min^−1^)	−6.18 ± 0.855	1.195 ± 0.268	−4.771 ± 1.538	2.147 ± 0.745	0.000	0.000	0.003	0.005	0.42	0.23	0.52
VCO_2_ (L·min^−1^)	−5.555 ± 0.717	0.905 ± 0.197	−3.412 ± 1.222	1.212 ± 0.48	0.000	0.000	0.006	0.013	0.12	0.55	0.53
RER	−10.742 ± 2.018	7.652 ± 1.871	−4.132 ± 3.867	3.027 ± 3.203	0.000	0.000	0.29	0.35	0.13	0.21	0.48
RR (breaths·min^−1^)	−6.15 ± 0.648	0.087 ± 0.015	−3.677 ± 1.002	0.087 ± 0.026	0.000	0.000	0.000	0.001	0.036	0.99	0.59
VT (l)	−5.259 ± 1.523	1.136 ± 0.638	−2.034 ± 3.016	0.705 ± 1.351	0.001	0.08	0.50	0.60	0.33	0.77	0.35
A-aDO_2_ tc (mmHg)	−4.313 ± 0.684	0.158 ± 0.057	−2.269 ± 2.081	0.116 ± 0.128	0.000	0.008	0.28	0.37	0.33	0.75	0.40

Nox, normoxia; Hyp, hypoxia; I, Intercept; S, slope; Ic, comparison of intercepts between normoxia and hypoxia; Sc, comparison of slopes between normoxia and hypoxia; HR, heart rate; VO_2_, oxygen uptake; VCO_2_, CO_2_ production; RER, respiratory exchange ratio; RR, respiratory rate; VT, tidal volume; P_E__T_co_2_, end-tidal Pco_2_; A-aDO_2_, alveolar-to-arterial oxygen pressure difference; tc, temperature corrected.

The intercept SE was 0.714 (*P* < 0.001) and slope SE 0.009 (*P* < 0.001).

**Figure 1:**
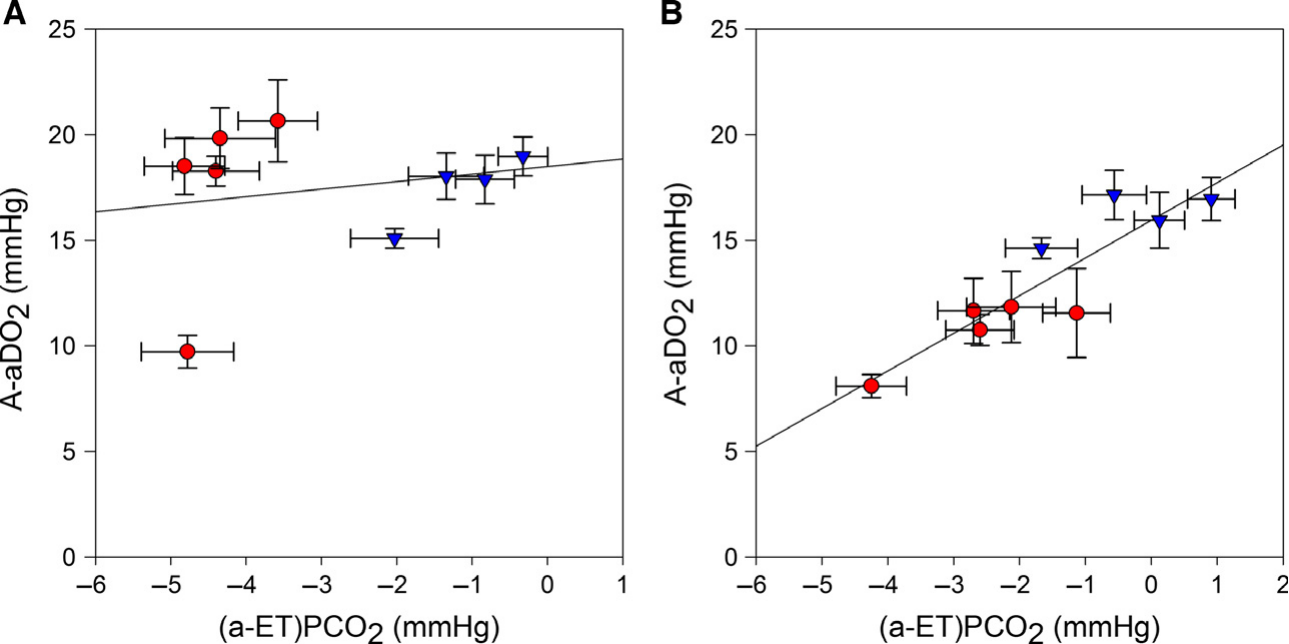
Relationship between alveolar-to-arterial O_2_ pressure difference (A-aDO_2_) and alveolar-to-end-tidal CO_2_ pressure difference ((a-ET)Pco_2_); (A) without correction of arterial blood gases for blood temperature and (B): after blood gases correction for blood temperature (A-aDO_2_ = 15.96 + 1.79 × (a-ET) Pco_2_; *r* = 0.90, EES = 1.45 mmHg, *n* = 9, each point representing the mean of 6–11 observations, *P* < 0.001). Error bars represent the standard error of the mean.

There was a close relationship between the mean (a-ET)Pco_2_ and the mean A-aDO_2_ when both F_I_O_2_ conditions were analyzed conjointly as follows:

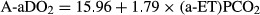
2

(*r* = 0.90, EES = 1.45 mmHg, *n* = 9, each point representing the mean of 6–11 observations, *P* < 0.001) (Fig.1).

## Discussion

In this study, we have shown that most of the negative (a-ET)Pco_2_ value is due to a lack of correction of P_a_co_2_ for blood temperature, and that a near maximal exercise intensity the mean (a-ET)Pco_2_ should be lying close to 0 mmHg in healthy humans. Moreover, we have demonstrated that in healthy humans, the temperature corrected (a-ET)Pco_2_ increases linearly with absolute and relative exercise intensity, VO_2_, VCO_2_, and RR in normoxia and hypoxia, with similar slopes in normoxia and severe hypoxia. Consequently, at the same absolute exercise intensity, the (a-ET)Pco_2_ is higher in hypoxia (i.e., less negative) than in normoxia. We have also shown that at a similar respiratory rate, (a-ET)Pco_2_ is higher in hypoxia than in normoxia indicating that factors other than, or in addition to, the respiratory rate or tidal volume should explain the greater (a-ET)Pco_2_ observed in hypoxia.

### Impact of temperature correction on the (a-ET)Pco_2_ difference

Since P_E__T_co_2_ overestimates P_a_co_2_ at all exercise intensities, the derived (a-ET)Pco_2_ has negative values as previously reported in young (Jones et al. 1979; Robbins et al. 1990; Liu et al. 1995; Williams and Babb 1997) and elderly men (St Croix et al. 1995). This study reveals the importance of correcting P_a_co_2_ for lung blood temperature has for the accurate determination of the (a-ET)Pco_2_. In fact, this correction alone explains ∼70% of the negative (a-ET)Pco_2_ at maximal exercise in normoxia and transforms the noncorrected negative (a-ET)Pco_2_ during maximal exercise in hypoxia to positive.

### Negative (a-ET)Pco_2_ values: fact or artifact?

In agreement with the previous investigators (Jones et al. 1979; Robbins et al. 1990; Liu et al. 1995; St Croix et al. 1995; Williams and Babb 1997), we have also observed negative (a-ET)Pco_2_ values during exercise, which increased with exercise intensity, as previously reported (Wasserman et al. 1967; Whipp and Wasserman 1969). It has been the subject of controversy whether negative (a-ET)Pco_2_ values really exist or if they result from multiple inaccuracies, including the use of different procedures to measure respiratory and blood gases (Forster 1977; Gurtner 1977; Scheid and Piiper 1980; Piiper 1986). In theory (a-ET)Pco_2_ negative values may be caused by several mechanisms acting conjointly or separately (for review see [Scheid and Piiper 1980; Stickland et al. 2013]).

In well-ventilated and perfused alveoli, the P_E__T_co_2_ represents the Pco_2_ during the phase of the respiratory cycle at which the P_A_co_2_ becomes closer to the mixed venous Po_2_. Thus, the P_E__T_co_2_ will always overestimate the actual P_A_co_2_ in well-ventilated and perfused alveoli. Underperfused alveoli have a rather low P_A_co_2_, which is even lower in areas that do not participate in gas exchange (dead space). Consequently, dead space ventilation may contribute to reduce P_E__T_co_2_ below mean P_A_co_2_, as observed at rest (Dubois et al. 1952). The increase in *V*t, VCO_2_, and mixed venous CO_2_ with exercise causes greater within-breath fluctuations of alveolar gas composition (Dubois et al. 1952) such that during expiration, P_A_co_2_ increases toward mixed venous Pco_2_ (P_v_co_2_) more rapidly as the increased CO_2_ production of exercise is evolved into a lung volume becoming smaller as expiration continues (Jones et al. 1979). This may result in P_E__T_co_2_ actually being higher than mean P_a_co_2_ during exercise (Jones et al. 1966). According to this description, we must have seen increasingly negative (a-ET)Pco_2_ with the increase of exercise intensity because the difference between P_v_co_2_ and P_a_co_2_ increases with exercise intensity. We actually observed the opposite, that is, (a-ET)Pco_2_ becomes less negative with the increase of exercise intensity. Our findings can be explained by several mechanisms. First, lack of Paco_2_ correction for arterial blood temperature as shown in this study.

Second, the increase in P_E__T_co_2_ with the exercise-induced widening of the intra-breath fluctuation in P_A_co_2_ is expected to be lower in severe hypoxia than in normoxia because the mixed venous Pco_2_ is lower while the inspiratory CO_2_ is similar to normoxia. Consequently, the magnitude of the mean P_E__T_co_2_ is lower in hypoxia and remains closer to the mean P_A_co_2_. Thus, the second mechanism agrees with a greater (less negative or more positive) (a-ET)Pco_2_ during exercise in severe hypoxia, as observed in the present study.

Third, lack or a very small right-to-left shunt may cause an elevation of (a-ET)Pco_2_ as P_a_co_2_ is expected to increase in proportion to the magnitude of the venous admixture (Whyte et al. 1993). Using the data generated in this study, we have estimated that during maximal exercise in normoxia, a 2% and 10% right-to-left shunt would increase P_a_co_2_ by 5 and 15 mmHg, respectively, even after accounting for the Haldane effect. In severe acute hypoxia, a 2% and 10% shunt will cause a 4 and 11 mmHg increase of P_a_co_2_, respectively. However, experiments using the multiple inert gas elimination technique have found no evidence of shunt during exercise (Hammond et al. 1986; Wagner et al. 1986; Hopkins et al. 1994, 1998). Although some passage of blood through arterial-venous anastomosis has been demonstrated in humans (Lovering et al. 2008, 2009), its magnitude is likely low. The fact that the (a-ET)Pco_2_ difference was negative or close to 0 mmHg concurs with a small or inexistent shunt in our experimental conditions. Moreover, shunt at maximal exercise has a greater impact on Paco_2_ than on Pao_2_ because the mixed venous CO_2_ content during exercise increases proportionally more than mixed venous O_2_ is reduced. Thus, a good correlation between the A-aDO_2_ and the A-aDCO_2_ is not expected with a high contribution of shunt to the impairment of pulmonary gas exchange during exercise because the shunt affects differently the A-aDO_2_ and the (a-ET)Pco_2_.

In summary, our results suggest that the negative (a-ET)Pco_2_ values observed in previous studies are likely due to lack of correction of P_a_co_2_ for blood temperature. The (a-ET)Pco_2_ difference is less negative during exercise in hypoxia than normoxia. At peak exercise, the mean Paco_2_ and the mean P_ET_co_2_ are similar, suggesting that P_E__T_co_2_ is a useful surrogate for P_a_co_2_. The mean (a-ET)Pco_2_ difference increases with exercise intensity and is closely related to the mean A-aDO_2_ difference. This is expected if similar mechanisms perturb the lung gas exchanges of O_2_ and CO_2_.
